# Impact of Preemptive vs. Conventional Tirofiban Administration on the Outcomes in Acute Branch Atheromatous Disease: A Propensity Score‐Weighted Cohort Study

**DOI:** 10.1002/cns.71120

**Published:** 2026-09-03

**Authors:** Chunying Li, Ying Jin, Xing Liu, Ying Liu, Weikang Yang, Yue Wang, Dongsheng Ju

**Affiliations:** ^1^ Department of Neurology Songyuan Jilin Oilfield Hospital Songyuan China; ^2^ Department of Magnetic Resonance Imaging Songyuan Jilin Oilfield Hospital Songyuan China

**Keywords:** branch atheromatous disease, multivariable regression, preemptive administration, propensity score weighting, tirofiban

## Abstract

**Background:**

Branch atheromatous disease (BAD) carries a high risk of early neurological deterioration (END). We evaluated the efficacy of preemptive tirofiban administration within 72 h compared with reactive rescue therapy following END.

**Methods:**

This retrospective, single‐center study enrolled 221 patients with BAD‐associated stroke. Based on the timing of tirofiban administration, patients were assigned to a preemptive intervention group (*n* = 127) or a conventional intervention group (*n* = 94). Preemptive intervention was defined as preemptive administration after clinical risk assessment, whereas conventional intervention was delayed until an increase of ≥ 2 points on the NIHSS score. Inverse probability of treatment weighting (IPTW) was applied to balance baseline covariates. Multivariable regression analysis was performed to identify independent predictors of an excellent 90‐day outcome, defined as a modified Rankin Scale (mRS) score ≤ 1.

**Results:**

After IPTW adjustment, the preemptive intervention group showed a significantly higher rate of excellent 90‐day outcomes (76.6% vs. 44.6%, *p* < 0.001) and a shorter median hospital stay (10 vs. 11 days, *p* = 0.02) compared with the conventional group. No between‐group differences were observed in mortality or stroke recurrence (*p* > 0.05). Early tirofiban use (adjusted OR = 4.411, 95% *CI*: 2.349–8.283) and a lower baseline NIHSS score (adjusted OR = 0.832, 95% *CI*: 0.707–0.980) independently predicted favorable outcomes. Subgroup analyses confirmed consistent benefits across most strata, with patients aged over 65 years deriving greater neuroprotection (*p* = 0.004 for age‐treatment interaction).

**Conclusion:**

Preemptive administration of tirofiban within the initial 72 h significantly improves 90‐day neurological outcomes and reduces hospital stay length in patients with BAD‐related stroke.

## Graphical abstract

In acute branch atheromatous disease stroke, preemptive tirofiban within 72 h significantly raised the 90‐day excellent outcome rate (76.6% vs. 44.6%) and shortened hospitalization without increasing mortality or recurrence. Preemptive administration independently predicted favorable recovery, with enhanced neuroprotection in patients over 65 years.

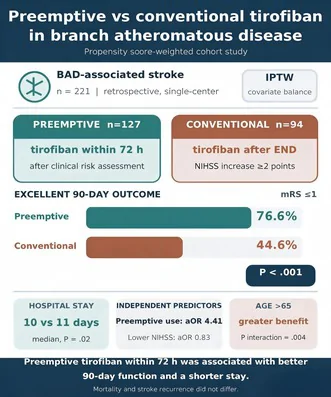

## 1 Introduction

Acute branch atheromatous disease (BAD) defines a distinct subtype of stroke [1, 2]. This condition results in infarction of the entire perfusion territory of the perforating artery, arising from stenosis or occlusion at the origin of the artery, or from plaque extending from the parent artery into the perforator's orifice. BAD frequently affects key vessels, such as the lenticulostriate arteries (LSA), paramedian pontine arteries (PSA), and anterior choroidal arteries (AChA) [3]. Clinically, its main presentation is early neurological deterioration (END), featuring progressive motor deficit (PMD), with an incidence reaching 30%–48%. This deterioration mostly occurs within 48 to 72 h of onset and is closely associated with poor prognostic outcomes and a high disability rate [4, 5, 6]. Data reveals that roughly 45% of patients suffering from BAD endure moderate to severe disability, as evidenced by a modified Rankin Scale (mRS) score of 3 or higher, 90 days following the onset of the condition [7]. Given its high progression and disability rates, BAD has emerged as a critical focal point and a significant challenge for clinical intervention.

Currently, intravenous thrombolysis is the primary method for restoring cerebral blood flow in patients with acute ischemic stroke (AIS). However, for patients with stroke associated with BAD, intravenous thrombolysis has limited efficacy in preventing END and cannot fully improve 90‐day outcomes [8, 9, 10]. Furthermore, owing to its limited therapeutic time window and a plethora of contraindications, it is suitable solely for a small subset of patients. Based on prior literary reports, in patients with BAD‐related stroke, elevated plasma levels of beta‐thromboglobulin and platelet factor 4 (PF4) are independent predictors of END [11], suggesting that active antiplatelet therapy has clinical benefits in preventing END. Aspirin and clopidogrel are clinically recommended for standard therapy, which can be administered as monotherapy or in combination. However, it is difficult to fully inhibit all relevant pathways of platelet aggregation [12], and some patients still experience END.

Tirofiban acts as a highly selective antagonist of the platelet glycoprotein IIb/IIIa receptor. It has a rapid onset of action and a short half‐life and holds promise as a superior alternative to traditional therapies [13]. The TREND study indicates that for patients with non‐cardioembolic stroke within 24 h of onset, tirofiban can reduce the risk of END without increasing the risk of bleeding [14]. A prospective study in China found that for patients with non‐cardiogenic stroke without large vessel occlusion, preemptive administration of tirofiban within 48 h of onset can effectively prevent the occurrence of END and promote early neurological improvement. Subgroup analysis suggested that patients with BAD derived even greater benefits [15]. However, there remains a dearth of high‐level support for the clinical benefits of preventive administration when compared to conventional remedial administration within a broader window period, such as within 72 h. Given this, the present study was designed as a retrospective cohort analysis, employing a doubly robust analytical model that combines inverse probability of treatment weighting (IPTW) with multivariate logistic regression. By extending the observation window to 72 h after onset, the study focuses on exploring the impact of early preemptive use of tirofiban on the excellent 90‐day functional outcome (mRS score of 0–1) in patients with acute BAD, aiming to provide more precise therapeutic decision‐making evidence for the clinical management of BAD.

## 2 Method

### 2.1 Research Subjects and Methods

A single‐center, retrospective cohort design was used for this study. Patients with BAD‐related stroke who sought medical treatment at Songyuan Jilin Oilfield Hospital from March 2023 to June 2025 were consecutively included.

Inclusion criteria: (1) Age ≥ 18 years old; (2) Onset within 72 h; (3) Good pre‐onset neurological function, defined as mRS score ≤ 1; (4) Presence of unilateral limb motor dysfunction of varying degrees, with 1 point ≤ unilateral limb motor score on the National Institutes of Health Stroke Scale (NIHSS) < 8 points; (5) Lesion on diffusion‐weighted imaging (DWI): Single (isolated) deep (subcortical) infarction; (6) The affected vessels included the LSA, PSA, or AChA, with infarction meeting at least one of the following DWI‐based criteria [16]: For LSA: On DWI, the infarct involved three or more horizontal axial slices within the corresponding perfusion territory, presenting either a fan‐shaped pattern extending from bottom to top or a comma‐like morphology; For PSA: On DWI, the infarction was contiguous with the ventral pontine surface, located unilaterally adjacent to the midline without crossing it; For AChA: DWI showed involvement of the posterior two‐thirds of the internal capsule's posterior limb. Exclusion criteria: (1) Imaging findings indicate stenosis of the responsible large vessel ≥ 50%; (2) Stroke resulting from other well‐defined causes, including immune‐mediated or infectious vasculitis, cardioembolic stroke, fat embolism, and abnormalities in platelet or coagulation function; (3) DWI evidence of cortical, watershed, or multiple cerebral infarctions; (4) Baseline cranial CT, MRI, MRA/CTA/DSA detection of any intracerebral hemorrhagic conditions, vascular malformations, aneurysms, brain abscesses, malignant space‐occupying lesions, or other non‐ischemic intracranial pathologies; (5) Patients who have received intravenous thrombolytic therapy; (6) Patients with incomplete follow‐up data or those lost to follow‐up.

### 2.2 Data Collection

Baseline and clinical information were obtained for all patients, covering demographic variables (gender, age), past medical history (hypertension, diabetes, coronary heart disease [CHD], hyperlipidemia, prior transient ischemic attack or stroke), lifestyle factors (smoking, alcohol use), admission NIHSS score, treatment duration, and baseline laboratory parameters (triglycerides, total cholesterol, low‐density lipoprotein cholesterol, high‐density lipoprotein cholesterol). Blood and other laboratory data were gathered within the first 24 h from admission.

### 2.3 Dosage and Administration

Drawing on the timing of tirofiban's clinical deployment, 221 patients were categorized into a preemptive intervention group and a conventional intervention group. Patients in the preemptive intervention group received standardized preemptive tirofiban therapy based on baseline DWI findings of corticospinal tract involvement, which indicates a high risk of limb paralysis progression and early neurological deterioration [17, 18, 19], to prevent symptom worsening. Corticospinal tract involvement was selected as the key trigger because it directly identifies the substrate of progressive motor deterioration in BAD, is readily assessable on admission DWI, and aligns with the thrombotic mechanism targeted by tirofiban. All baseline DWI images were independently assessed by two blinded neuroradiologists, with disagreements resolved by a senior radiologist. In contrast, in the conventional intervention group, tirofiban treatment was only initiated upon the manifestation of clear signs of disease progression and worsening, specifically defined as an increase of ≥ 2 points in the NIHSS score relative to the baseline level. Specific usage of tirofiban: The drug was given intravenously at an initial rate of 0.4 μg/kg/min over 30 min, after which a maintenance infusion was continued at 0.1 μg/kg/min for 48 to 72 h. After completion of the infusion, standardized sequential antiplatelet therapy was initiated. Other treatments are managed in accordance with international and Chinese guidelines [20, 21]. All treatment plans were jointly determined by two senior neurologists after a thorough assessment of the risk–benefit ratio, and full informed consent was obtained from the patients or their guardians.

### 2.4 Outcome Evaluation and Observation Indicators

The primary endpoint was an excellent 90‐day prognosis, defined as a mRS score ≤ 1. Secondary endpoints comprised a favorable prognosis (mRS 0–2) and hospital stay duration. Based on medical records and imaging, we recorded the 90‐day incidence of symptomatic intracranial hemorrhage and all‐cause mortality. Neurological recovery was assessed by the mRS performed 90 days after stroke onset, through outpatient or telephone follow‐up conducted by physicians who were blinded to treatment assignment.

### 2.5 Statistical Analysis

All statistical analyses were conducted utilizing R software (version 4.5.1). For measurement data, the Kolmogorov–Smirnov test was initially applied to assess the normality of their distribution. Continuous data for those with a normal distribution were shown as the mean ± standard deviation (*x* ± *s*), and group comparisons were analyzed using the two independent samples *t*‐test. Data that did not follow a normal distribution, such as NIHSS scores, were shown as median and interquartile range (IQR) and were analyzed for group differences by the Mann–Whitney U test. Count data were shown as number and percentage (%), and comparisons were made by the Pearson chi‐square test or Fisher's exact test according to corresponding appropriate circumstances.

To minimize potential selection bias and confounding inherent in the retrospective design, stabilized IPTW was employed. First, we estimated the propensity score of each patient receiving preemptive tirofiban treatment with a multivariable logistic regression model including all baseline covariates. Second, we calculated stabilized weights to estimate the ATE in the population, without interference of extreme weights on the point estimate of treatment efficacy, while retaining the same sample size as the original study.

Effectiveness of the weighting process was quantitatively evaluated by using the standardized mean difference (*SMD*). We defined *SMD* < 0.1 as ideal covariate balance between groups and this was illustrated using a Love plot. In the final outcome analysis, excellent functional prognosis at 90 days was the dependent variable. An IPTW‐weighted logistic regression model was constructed to assess the independent effect of preemptive intervention. This study presents the adjusted odds ratio (OR), 95% CI, and *p* values associated with that effect. Robust standard errors were applied uniformly to correct for variance issues when weighting and were done to ensure the validity of statistical inferences. An unweighted multivariable regression analysis was conducted as a sensitivity analysis to validate the robustness of the primary findings.

## 3 Results

### 3.1 Flow of Study Subjects and General Information

**FIGURE 1:**
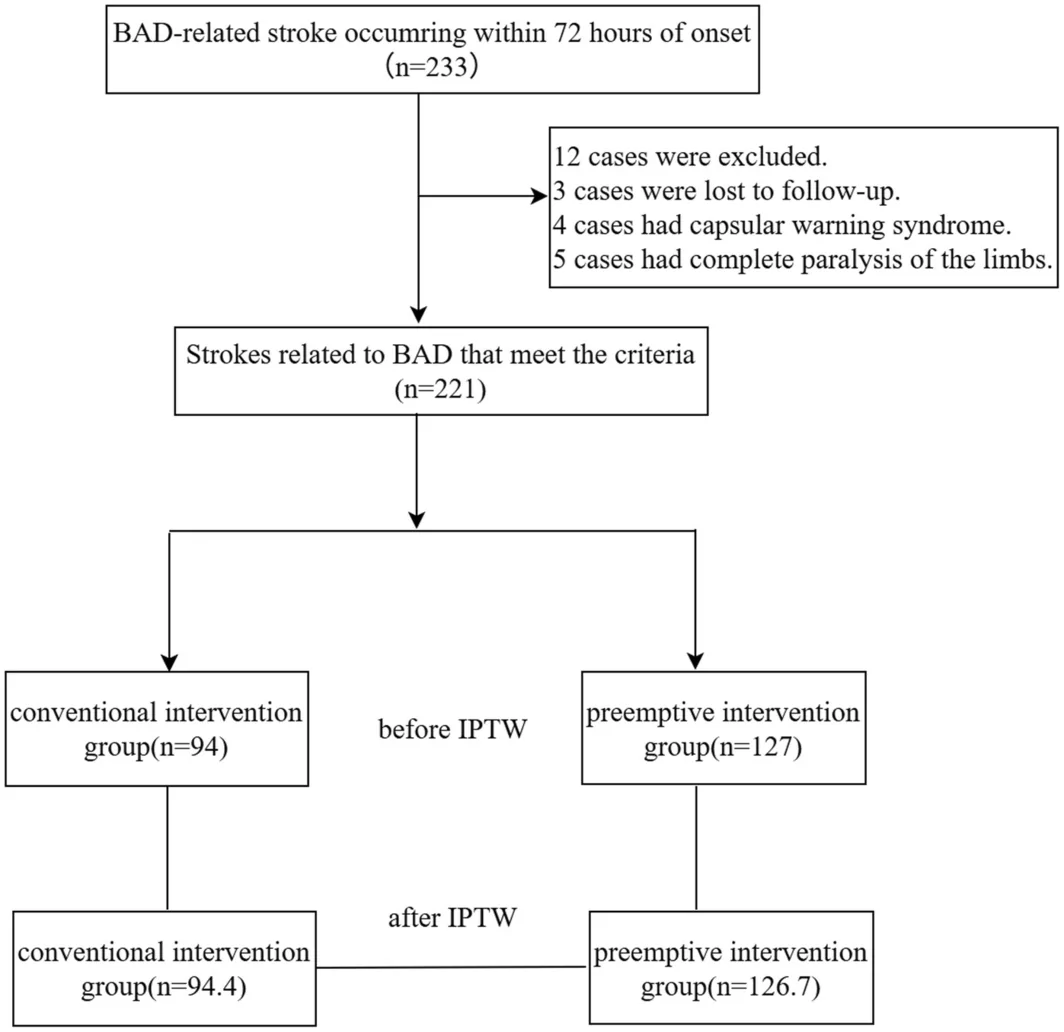
Patient selection flowchart. Of 233 patients with branch atheromatous disease‐related stroke screened within 72 h of onset, 221 were included after exclusion of 12 patients. Patients were assigned to the conventional intervention group or preemptive intervention group before and after inverse probability of treatment weighting. BAD, branch atheromatous disease; IPTW, inverse probability of treatment weighting.

The initial study population, comprising 233 patients with acute stroke arising from BAD, served as the baseline cohort. Of these, three patients were excluded because of loss to follow‐up, and another nine were excluded owing to non‐fulfillment of the inclusion criteria. The detailed workflow is presented in Figure 1.

Of the 221 patients included, 68 (30.8%) were female. The median age at admission was 64 years (IQR: 58–72), and the median NIHSS score was 3 (IQR: 2–5). Patients were assigned to the preemptive intervention group (*n* = 127, 57.5%) or the conventional intervention group (*n* = 94, 42.5%).

### 3.2 Evaluation of the Effectiveness and Balance of Propensity Score Weighting

**FIGURE 2:**
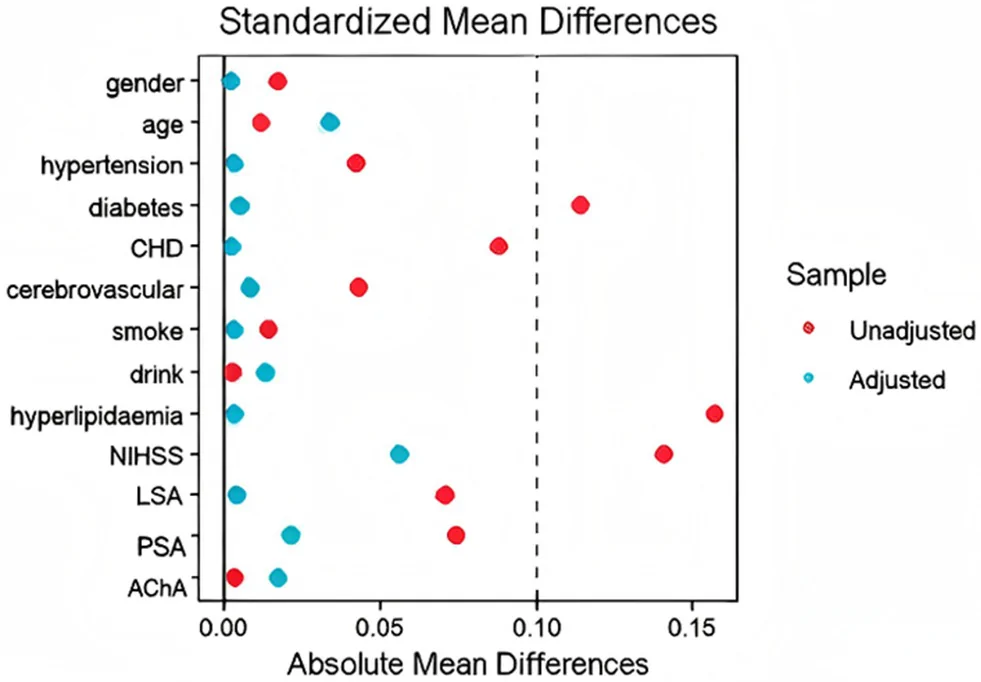
Covariate balance before and after inverse probability of treatment weighting. Absolute standardized mean differences are shown before and after weighting, with 0.10 indicating the threshold for acceptable balance. CHD, coronary heart disease; NIHSS, National Institutes of Health Stroke Scale; SMD, standardized mean difference; LSA, lenticulostriate arteries; PSA, paramedian pontine arteries; AChA, anterior choroidal arteries.

This study sequentially enrolled a total of 221 patients with acute BAD. Notably, baseline covariate imbalance was detected in the original cohort: hyperlipidaemia differed significantly between the preemptive and conventional intervention groups. After applying stabilized IPTW, *SMD* analysis revealed that the *SMD*s of all baseline covariates significantly decreased to below 0.1 after weighting (Table 1). The visual results from the Love plot (Figure 2) further confirmed that the baseline profiles between the two groups achieved a relatively ideal statistical balance, laying a reliable foundation for the subsequent conclusions.

**TABLE 1:** Baseline and clinical characteristics of two groups before and after propensity score weighting.

Characteristics	Before propensity score weighting				After propensity score weighting				
	Conventional *n* = 94	Preemptive *n* = 127	Test value	*p*	Conventional *n* = 94.4	Preemptive *n* = 126.7	Test value	*p*	*SMD*
Age, M (Q_1_, Q_3_)	65 (58,73)	63 (58,72)	−0.408	0.68	64.4 (57.0,72.1)	63.0 (57.9,71.5)	−0.269	0.79^b^	0.033
NIHSS,M (Q_1_, Q_3_)	3 (2, 5)	3 (2, 5)	−1.226	0.22	3 (2,5)	3.0 (2.0,4.3)	0.154	0.88^b^	0.052
Male, *n* (%)	66 (70.2)	87 (68.5)	0.074	0.79	66 (69.9)	88.4 (69.8)	0.001	0.98^a^	0.003
Hypertension, *n* (%)	75 (79.8)	96 (75.6)	0.543	0.46	72.3 (76.5)	97.4 (76.8)	0.002	0.96^a^	0.007
CHD, *n* (%)	26 (27.7)	24 (18.9)	2.369	0.12	21.2 (22.4)	28.7 (22.6)	0.002	0.97^a^	0.005
Diabetes, *n* (%)	44 (46.8)	45 (35.4)	2.906	0.09	38.2 (40.4)	50.6 (39.9)	0.005	0.94^a^	0.010
Cerebrovascular, *n* (%)	24 (25.5)	27 (21.2)	0.555	0.46	20.4 (21.6)	28.3 (22.4)	0.020	0.89^a^	0.019
Smoke, *n* (%)	42 (44.7)	55 (43.3)	0.041	0.84	42.1 (44.6)	57.0 (45.0)	0.002	0.96^a^	0.007
Drink, *n* (%)	36 (38.3)	49 (38.6)	0.002	0.97	39.0 (41.3)	57.7 (40.0)	0.034	0.85^a^	0.027
Hyperlipidaemia, *n* (%)	23 (24.5)	51 (40.2)	5.970	0.02	31.9 (33.8)	42.4 (33.4)	0.002	0.97^a^	0.007
VascularSegmentation, *n* (%)			1.525	0.47			0.051	0.95^a^	0.046
LSA	34 (36.2)	37 (29.1)			30.7 (32.5)	40.7 (32.1)			
PSA	36 (38.3)	58 (45.7)			37.1 (39.3)	52.5 (41.4)			
AChA	24 (25.5)	32 (25.2)			26.6 (28.2)	33.6 (26.5)			

Abbreviations: AChA, anterior choroidal arteries; CHD, coronary heart disease; LSA, lenticulostriate arteries; NIHSS, National Institutes of Health Stroke Scale; OR, odds ratio; PSA, paramedian pontine arteries; SMD, standardized mean difference.

^a^ The χ2 test statistic.

^b^ The Z test statistic.

### 3.3 The Impact of Preemptive Administration of Tirofiban on Primary and Secondary Outcomes

**FIGURE 3:**
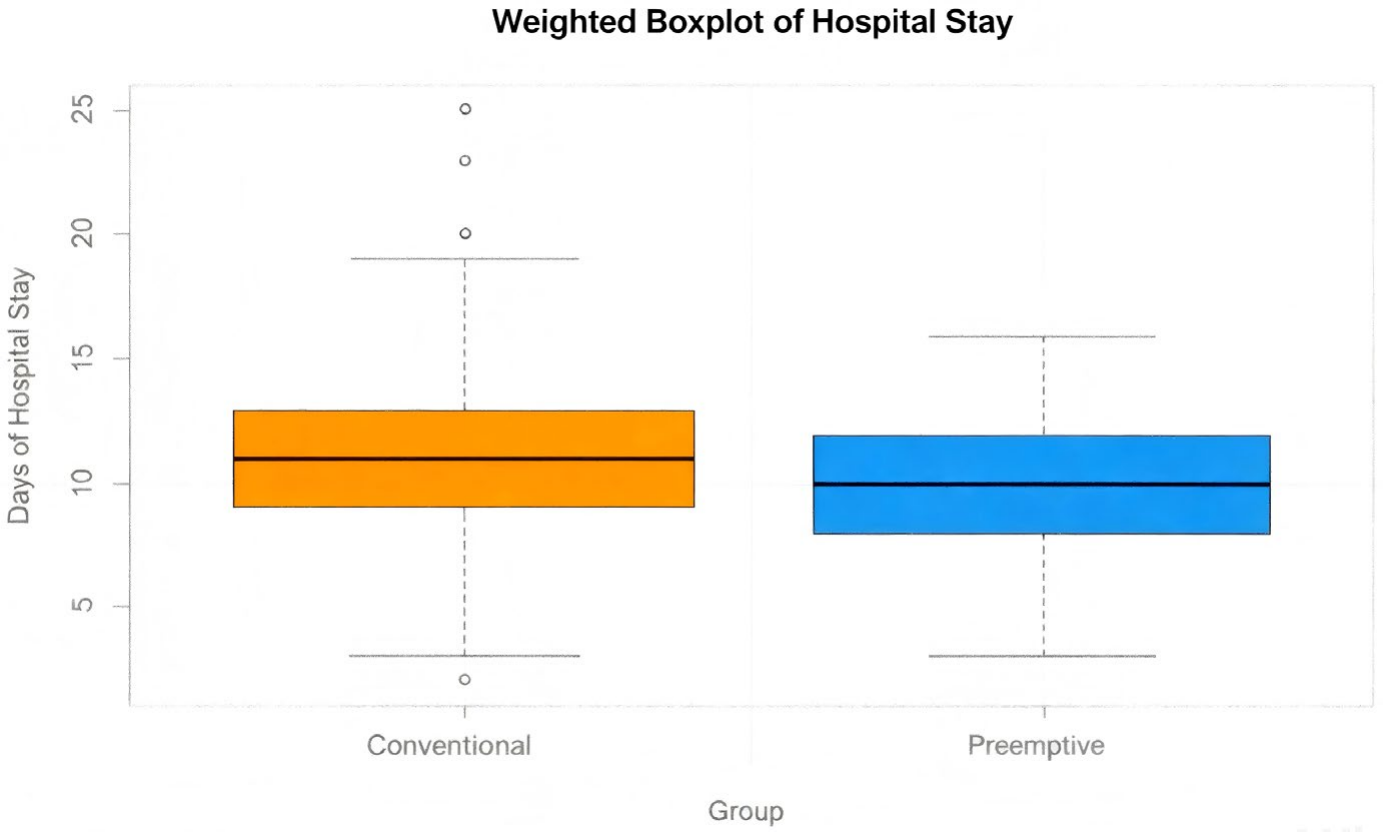
Weighted hospital stay duration by treatment group. The box plot shows the distribution of hospital stay after inverse probability of treatment weighting in the conventional and preemptive intervention groups. IPTW, inverse probability of treatment weighting.

Primary efficacy and safety outcomes are summarized in Table 2. Following propensity score weighting, the proportion of patients achieving an excellent 90‐day prognosis was significantly greater in the preemptive intervention group than in the conventional intervention group (97.1/126.7 [76.6%] vs. 42.1/94.4 [44.6%], *p* < 0.001). As shown in the box plot (Figure 3) and Table 2, the duration of hospital stay was notably shorter for the preemptive intervention group compared to the conventional intervention group, with values of [10 (8–12) days vs. 11 (9–13) days, *Z* = −2.376, *p* = 0.02]. There were no statistically significant differences in the 90‐day post‐onset mortality rate and stroke recurrence rate between the two groups (both *p* > 0.05). No symptomatic intracranial hemorrhage occurred during the observation period in either group. The findings indicate that preemptive intervention not only enhances neurological function but also holds potential health economic benefits.

**TABLE 2:** Efficacy and safety endpoints.

Endpoints	Preemptive, *n* = 126.7	Conventional, *n* = 94.4	Test value	*p*
90d excellent prognosis, *n* (%)	97.1 (76.6)	42.1 (44.6)	21.566^a^	< 0.001
90d favorable prognosis, *n* (%)	110.1 (86.9)	55.3 (58.6)	22.004^a^	< 0.001
Duration, M (Q_1_, Q_3_)	10 (8,12)	11 (9,13)	−2.376^b^	0.02
90d stroke recurrence	1.6 (1.2)	1.1 (1.2)	0.001	0.98
90d post‐onset mortality	0.7 (0.6)	1.8 (1.9)	1.070	0.30

^a^ The χ2 test statistic.

^b^ The Z test statistic.

### 3.4 Analysis of Influencing Factors for 90‐Day Excellent Prognosis Following Propensity Score Weighting

As the primary analysis, inverse probability of treatment weighting was applied to balance baseline covariates and evaluate the association between preemptive tirofiban and 90‐day excellent prognosis. Weighted univariate and multivariable logistic regression analyses were further performed to explore independent predictors and address residual confounding. Weighted univariate analysis identified admission NIHSS score and preemptive tirofiban as significant factors associated with the outcome (Table 3). The weighted multivariable model, including variables with *p* < 0.1 from univariate analysis plus prespecified confounders (age and sex), indicated that both preemptive tirofiban (adjusted OR = 4.411, 95% CI: 2.349–8.283, *p* < 0.001) and lower admission NIHSS score (adjusted OR = 0.832, 95% CI: 0.707–0.980, *p* = 0.03) were independent predictors of excellent 90‐day prognosis.

**TABLE 3:** Unweighted and IPTW‐weighted logistic regression analyses for excellent 90‐day outcome.

Characteristics	Before propensity score weighting						After propensity score weighting					
	Univariate analysis			Multivariate analysis			Univariate analysis			Multivariate analysis		
	OR	95% CI	*p*	OR	95% CI	*p*	OR	95% CI	*p*	OR	95% CI	*p*
preemptive intervention	4.003	2.248~7.129	< 0.001	4.557	2.409~8.619	< 0.001	4.061	2.208~7.466	< 0.001	4.411	2.349~8.283	< 0.001
Male	0.612	0.331~1.129	0.12	0.603	0.282~1.290	0.19	0.581	0.302~1.119	0.10	0.488	0.225~1.055	0.07
Age	0.994	0.967~1.021	0.66	0.992	0.959~1.025	0.62	0.988	0.960~1.017	0.40	0.975	0.943~1.008	0.14
Hypertension	0.665	0.338~1.311	0.24				0.844	0.410~1.741	0.65			
Diabetes	0.787	0.452~1.371	0.40				0.944	0.525~1.698	0.85			
CHD	1.063	0.552~2.048	0.85	1.346	0.643~2.818	0.43	1.358	0.680~2.712	0.39			
Cerebrovascular	0.525	0.278~0.990	0.05				0.606	0.315~1.204	0.16			
Smoke	0.730	0.421~1.264	0.26				0.736	0.412~1.315	0.30			
Drink	0.754	0.432~1.318	0.32				0.627	0.349~1.129	0.12			
Hyperlipidaemia	1.630	0.897~2.962	0.11				1.231	0.648~2.337	0.52			
NIHSS	0.841	0.741~0.954	0.01	0.811	0.704~0.935	0.004	0.854	0.722~1.010	0.07	0.832	0.707~0.980	0.03
LSA	As a reference						As a reference					
PSA	1.068	0.562~2.030	0.84				0.991	0.502~1.955	0.98			
AChA	0.829	0.404~1.701	0.61				0.734	0.343~1.571	0.42			

Abbreviations: AChA, anterior choroidal arteries; CHD, coronary heart disease; LSA, lenticulostriate arteries; NIHSS, National Institutes of Health Stroke Scale; OR, odds ratio; PSA, paramedian pontine arteries.

As a sensitivity analysis to verify result robustness, we performed unweighted univariate and multivariable regression on the original cohort. Results were highly consistent with the weighted analyses: preemptive tirofiban and admission NIHSS score remained independently associated with the outcome, confirming stable conclusions across different analytical strategies.

### 3.5 Subgroup Analysis Results

**FIGURE 4:**
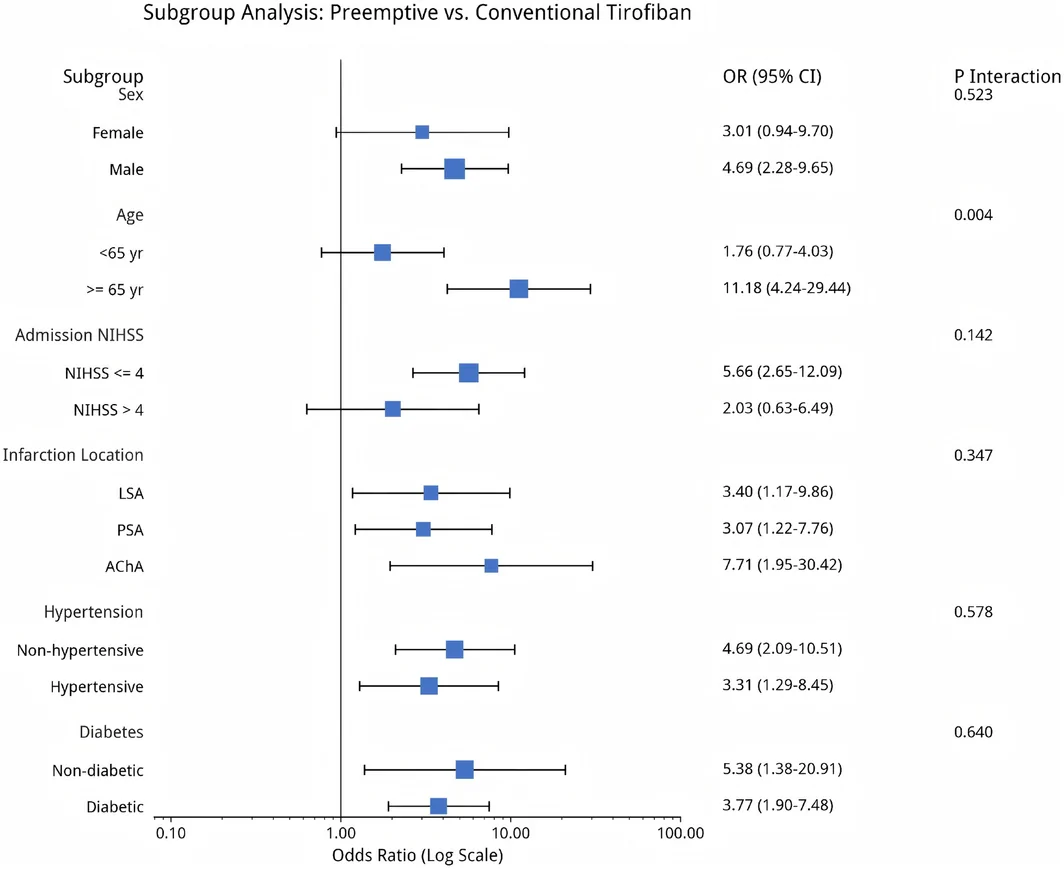
Subgroup analysis of excellent 90‐day outcome. Odds ratios and 95% confidence intervals compare preemptive tirofiban with conventional intervention across prespecified subgroups; odds ratios greater than 1 favor preemptive tirofiban. CI, confidence interval; NIHSS, National Institutes of Health Stroke Scale; OR, odds ratio; LSA, lenticulostriate arteries; PSA, paramedian pontine arteries; AChA, anterior choroidal arteries.

Subgroup analysis further confirmed that the benefits of preemptive tirofiban treatment remained highly consistent across most strata, including gender, vascular territory, pre‐existing diabetes, antecedent hypertension, and baseline NIHSS score. Notably, the study revealed a significant interaction between age and treatment strategy (*p* = 0.004). Although patients of all age groups show a trend of benefit, elderly patients aged over 65 derive significantly greater neuroprotective effects and improvements in prognosis from early preemptive use of tirofiban compared to younger patients. Stratified subgroup results with covariate interaction tests are shown as a forest plot in Figure 4.

## 4 Discussion

BAD has progressively gained recognition as a pivotal cause of acute subcortical infarction, notably due to its strong association with END and an elevated risk of stroke recurrence, particularly involving progressive motor deficits [1]. This study is a single‐center, retrospective investigation. Following propensity score weighting analysis, early preemptive tirofiban was found to significantly increase the percentage of acute BAD‐related stroke patients achieving an excellent 90‐day outcome (mRS ≤ 1) when given within 72 h of stroke onset. This finding underscores the feasibility of extending the treatment window for tirofiban to 72 h. An mRS score of ≤ 1 signifies that the patient experiences no significant neurological disability, is capable of independently performing daily activities, and has largely regained social functionality [22]. Taken together, these findings suggest that using tirofiban early as part of intensified antiplatelet therapy may represent an effective strategy for improving long‐term neurological function and quality of life in high‐risk BAD patients.

Tirofiban is a potent and reversible glycoprotein GPIIb/IIIa receptor inhibitor, which exerts pharmacological effects by interfering with fibrinogen binding to platelet GPIIb/IIIa receptors, reducing platelet aggregation and cross‐linking [23, 24]. Accumulated preclinical evidence shows tirofiban has additional pleiotropic effects in ischemic stroke models, including reducing pro‐inflammatory cytokines (IL‐1β, IL‐6, TNF‐α) via microglial regulation, attenuating oxidative stress (SOD up‐regulation, MDA reduction), and mitigating neuronal apoptosis [13, 25, 26]. Genomic analyses link these effects to neurotrophic factor and inflammatory gene regulation [24]. These pathways have only been validated in preclinical settings, not in our clinical cohort. Combined with published basic research evidence, we speculate that preemptive tirofiban reduces END risk and improves 90‐day neurological function primarily through two complementary pathways: first, it potently inhibits platelet aggregation and restricts thrombus expansion on unstable atherosclerotic plaques via its well‐established GPIIb/IIIa receptor blocking effect; second, it may indirectly alleviate secondary inflammatory and oxidative injury in the ischemic penumbra through the pleiotropic effects observed in preclinical studies. Nevertheless, these mechanistic explanations remain speculative inference based on existing basic research, and dedicated translational clinical studies are needed for verification in BAD patients.

Consistent with well‐established clinical consensus, a lower admission NIHSS score independently predicted excellent 90‐day functional outcomes in this BAD cohort. This suggests that in clinical practice, baseline NIHSS score combined with tirofiban administration timing may serve as a practical reference to support individualized antiplatelet treatment decision‐making.

The length of hospital stay in the preemptive intervention group was significantly shorter than that in the conventional intervention group, suggesting that early preemptive administration of tirofiban can shorten patients' hospital stays by rapidly controlling the condition and reducing the risk of neurological deterioration. This approach not only alleviates the physical and mental suffering as well as the economic burden of patients but also enhances the utilization efficiency of medical resources, demonstrating good clinical practicality and health economic value. Moreover, it does not increase the risk of fatal adverse events or bleeding, reflecting a favorable benefit–risk ratio of this treatment strategy.

The interaction within age subgroups indicates that age may be an important effect modifier influencing the therapeutic efficacy of tirofiban. That is, there are age‐related differences in the benefits of early preemptive use of tirofiban. Moreover, the results of this study show that the OR value is higher in the subgroup of patients aged ≥ 65 years, suggesting that elderly patients (≥ 65 years old) experience more significant improvements in prognosis from early preemptive use of tirofiban. The age‐related disparity in benefits could be intricately linked to the clinicopathological features of elderly patients [27]: Elderly patients (aged ≥ 65 years) often have multiple underlying diseases. The degree of atherosclerosis in their blood vessel walls is more severe, and the plaques are more fragile. This is related to the fact that both the vascular volume and diameter, as well as the intimal thickness, increase with age, while the tissue's healing and repair capabilities decline with age. These changes result in a reduction in elastin fibers, smooth muscle cells, and the total number of cells, along with an increase in lipid, cholesterol, calcium phosphate deposition, and angiogenesis. Elderly patients have a significantly higher risk of END and vascular reocclusion compared to younger patients [28]. A higher odds ratio underscores that early preemptive tirofiban works more prominently to avert disease deterioration and enhance prognosis, acting via fast platelet aggregation blockade, inhibition of thrombus progression, and protection of the ischemic penumbra. For younger patients (age < 65 years), baseline vascular condition is typically superior and plaques are relatively stable, which is accompanied by a diminished likelihood of disease progression. As a result, the preventive advantages of tirofiban are comparatively limited in this demographic, yielding an OR lower than that observed in the elderly subgroup. This result still requires further verification. Subsequent studies could expand the sample size and conduct detailed analyses on the optimal timing of administration, dosage, and duration of treatment with tirofiban in the elderly subgroup aged ≥ 65 years, with a focus on verifying its safety and efficacy.

Several limitations of the present study should be acknowledged. While stabilized IPTW was employed to emulate a randomized environment, the potential for unmeasured confounding remains a limitation of this retrospective design. All study subjects were derived from a single‐center clinical setting, which restricts the generalizability of the findings. Additionally, a relatively limited cohort size may result in insufficient statistical power for subgroup analyses. Furthermore, routine follow‐up imaging was not performed in all stable patients, which may underdetect asymptomatic hemorrhagic transformation, cause potential ascertainment bias, and limit safety conclusions to symptomatic intracranial hemorrhage. Finally, this study failed to conduct an analysis and discussion on the dosage and administration duration of tirofiban. Subsequent studies could adopt a prospective, multicenter, randomized controlled trial design and expand the sample size to enhance statistical power.

In summary, through a retrospective cohort study design and by employing methods such as propensity score weighting, multivariate logistic regression, and subgroup analysis, our analysis additionally confirmed that early preemptive administration of tirofiban can significantly increase the rate of excellent 90‐day outcomes in high‐risk patients with BAD, shorten the duration of hospitalization, and does not increase the rates of symptomatic intracranial hemorrhage, mortality, or stroke recurrence. It exhibits a favorable benefit–risk ratio and health economic value. Preemptive administration of tirofiban and a lower admission NIHSS score were further identified as independent predictors of an excellent 90‐day prognosis. A significant interaction was observed only for age, suggesting greater benefit among patients aged ≥ 65 years. The findings of this study provide preliminary clinical evidence for the early antiplatelet strategy in patients with BAD‐related stroke, and may serve as a reference for real‐world clinical decision‐making, as well as a foundational basis for designing future large‐scale multicenter prospective studies.

## Funding

The authors have nothing to report.

## Ethics Statement

This study was conducted in strict accordance with the ethical principles set forth in the Declaration of Helsinki. The study protocol was reviewed and approved by the Clinical Research Ethics Committee of Songyuan Jilin Oilfield Hospital (No.:XSLW‐LLSCPJ‐001). Signed informed consent was obtained from all included patients or their family members prior to diagnosis and treatment.

## Conflicts of Interest

The authors declare no conflicts of interest.

## Data Availability

The data supporting the conclusions of this study are available from the corresponding author upon reasonable request.
